# Drosophila microRNAs 263a/b Confer Robustness during Development by Protecting Nascent Sense Organs from Apoptosis

**DOI:** 10.1371/journal.pbio.1000396

**Published:** 2010-06-15

**Authors:** Valérie Hilgers, Natascha Bushati, Stephen M. Cohen

**Affiliations:** 1 Temasek Life Sciences Laboratory, National University of Singapore, Singapore; 2 PhD Programme, European Molecular Biology Laboratory, Heidelberg, Germany; 3 Department of Biological Sciences, National University of Singapore, Singapore; University of Wisconsin, United States of America

## Abstract

*miR-263a/b* are members of a conserved family of microRNAs that are expressed in peripheral sense organs across the animal kingdom. Here we present evidence that *miR-263a* and *miR-263b* play a role in protecting Drosophila mechanosensory bristles from apoptosis by down-regulating the pro-apoptotic gene *head involution defective*. Both microRNAs are expressed in the bristle progenitors, and despite a difference in their seed sequence, they share this key common target. In *miR-263a* and *miR-263b* deletion mutants, loss of bristles appears to be sporadic, suggesting that the role of the microRNAs may be to ensure robustness of the patterning process by promoting survival of these functionally specified cells. In the context of the retina, this mechanism ensures that the interommatidial bristles are protected during the developmentally programmed wave of cell death that prunes excess cells in order to refine the pattern of the pupal retina.

## Abstract 2

microRNA-263a/b confer robustness to sense organ development by controlling expression of the pro-apoptotic gene hid during apoptotic tissue pruning in *Drosophila*.

## Author Summary

In spite of continuous challenges from the ever-changing environment, biological systems exhibit incredible stability in their developmental and physiological processes. In addition to extrinsic variability caused by environmental fluctuations, cells face intrinsic variability arising from the inherent noise of gene expression and of other molecular processes. microRNAs, which act as post-transcriptional regulators of gene expression, are beginning to be recognized for their ability to confer robustness to biological systems by buffering the effects of noisy gene expression. Although noise often is viewed as destabilizing, some biological processes make use of noise in order to make stochastic decisions. In this paper, we describe a role for microRNAs in preventing the stochastic elimination of excess cells in the developing fly retina. After the sense organs that make up the eye have been specified, pruning of excess cells occurs through the action of the gene *hid*, the expression of which triggers cell death. Specific mechanisms are needed to protect specialized cells which need to be maintained to ensure that only excess cells are eliminated. We report that a pair of related microRNAs, *miR-263a/b*, protect sense organs during this pruning process by directly acting upon and limiting the expression of the proapoptotic gene *hid*. This example, illustrates a novel function for miRNAs in ensuring developmental robustness during apoptotic tissue pruning.

## Introduction

Organogenesis requires the organization of different cell types into precise spatial patterns. The Drosophila compound eye has proven to be a useful model system in which to investigate how such ordered patterns are established and maintained. The mature retina consists of ∼750 regular units, called ommatidia. Each ommatidium consists of eight photoreceptors, four cone cells, and two primary pigment cells. Individual ommatidia are separated by a layer of secondary and tertiary pigment cells. The “interommatidial” lattice also includes sense organs called interommatidial bristles (IOB). The IOB are mechanosensory hair cells, which may help the fly to avoid damage to the eye surface. IOB develop from a distinct set of sensory organ precursors (SOP), specified at discrete positions among the array of interommatidial cells [1].

In the developing eye imaginal disc, a field of naïve cells is produced by proliferation and the requisite number of ommatidial precursors is selected in a process of spatially patterned cell-type specification [2]. Short-range signaling by the initially specified R8 photoreceptor cell determines the fate of surrounding cells to make the full complement of neuronal cells needed for the ommatidium. Accessory cells, such as pigment cells, are then selected from the surrounding field of interommatidial cells. As in most developing neuronal systems, progenitor cells are over-produced, and excess cells eliminated by apoptosis after the correct pattern has been generated. In the eye imaginal disc, excess interommatidial cells are removed by two waves of programmed cell death during early pupal stages to produce the near-perfect array of ommatidia found in the adult eye [3],[4]. A patterning process based on “pruning” of excess cells requires a mechanism to protect important functionally specified cells.

Mechanisms to ensure robustness are an important feature of developmental systems that can be subject to perturbation. MicroRNAs (miRNAs) have been proposed to play a role in conferring robustness during development [5],[6]. This is exemplified by *miR-7*, which has been shown to contribute to the robustness of regulatory networks that ensure correct sense organ specification in Drosophila [7]. Although *miR-7* is not required under normal conditions, SOP patterning was compromised when *miR-7* mutant flies were subjected to environmentally challenging conditions. miRNAs act as post-transcriptional regulators that limit levels of target gene expression. This property makes them well suited to buffer fluctuating levels of gene activity. It may also make them well suited to serve a protective function during patterned tissue pruning.

In this report we present evidence that the *miR-263a/b* family of miRNAs contributes to the robustness of sense organ development. During apoptotic tissue pruning, functionally specified cells such as photoreceptors and mechanosensory organs are protected, while excess cells are eliminated. Mechanisms to ensure survival of specific cells are needed. Tissue pruning in the developing retina depends on activity of the pro-apoptotic gene *hid* [8], however the mechanisms that govern the decision as to which cells are lost are not fully understood. In the absence of *miR-263a/b* sensory bristles are lost, like other cells, in a stochastic manner. Through a process of experimental validation we identify *hid*, among over 50 candidates examined in vivo, as a biologically important target of *miR-263a/b* in this context. While *hid* and other proapoptotic genes are targeted by other miRNAs, including *bantam* and the *miR-2* family [5],[9],[10], none of these interactions has been shown to affect apoptotic pruning. Thus *miR-263a/b* may have a dedicated antiapoptotic role to ensure the robustness of sense organ development in a fluctuating developmental landscape.

## Results

### Loss of Sense Organs in Flies Lacking *miR-263a* and *miR-263b*

**Figure 1:**
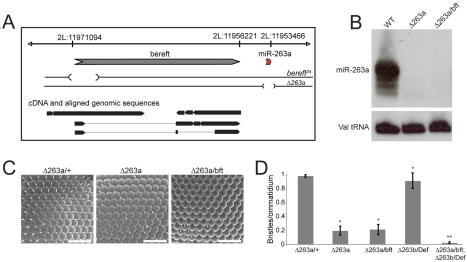
*miR-263a* and *miR-263b* mutants. (A) Schematic representation of the *bereft*/*miR-263a* locus. *miR-263a* is located 2.7 Kb downstream of the 3′ end of the annotated *bereft* transcript. The extent of the 2.8 Kb deletion in the *bereft^24^* allele and that of the 350 nt deletion in the *miR-263a* deletion allele (Δ263a) are indicated. (B) Northern blot showing mature *miR-263a* RNA levels in total RNA extracted from adult control flies (WT) and two combinations of *miR-263a* mutant alleles. The most abundant product of miR-263a detected by sequencing is 24 nt in length [25]. Δ263a denotes the targeted *miR-263a* deletion, *bft* denotes the *bft^24^* allele described in [11]. A probe for Valine tRNA was used to monitor loading. (C) Scanning electron micrographs (SEM) of adult eyes from *miR-263a/+* heterozygous control and *miR-263a* mutant flies. + denotes the wild-type chromosome. Scale bars = 50 µm. (D) Quantification of IOB in *miR-263a* and *miR-263b* single mutants and *miR-263a miR-263b* double mutant flies. “Def” denotes *Df(3L)X-21.2*. Error bars represent mean ± SD. *N* = 10–30 flies per genotype. [*] and [**] = *p*<0.001 using two-tailed unpaired Student's *t* test comparing to the *miR-263a/+* control [*] or the *miR-263a* single mutant [**].

*miR-263a* is located near the *bereft* locus on chromosome 2L (Figure 1A). cDNA evidence has indicated that *bereft* encodes a spliced transcript, however, one without an obvious protein-coding region [11]. Expression of this cDNA in transgenic flies did not rescue the defects that were attributed to *bereft* mutants [11]. In this light, we asked if *miR-263a* might be the functional product of the *bereft* locus. To address this, ends-out homologous recombination was used to generate a small deletion removing *miR-263a*. Three hundred and fifty nucleotides including the miRNA hairpin were replaced with a mini-white gene cassette (Figure 1A) [12]. The absence of the mature *miR-263a* miRNA was confirmed by Northern blot using total RNA isolated from adult flies homozygous mutant for the targeted allele (*Δ263a*, Figure 1B). Mature *miR-263a* was also missing in flies carrying the *bereft^24^* allele in trans to the *Δ263a* deletion allele (*Δ263a/bft*, Figure 1B), as well as in other *bereft* mutants (Figure S1). The *bereft^24^* allele is a 2.8 kb deletion that removes the first exon of the *bereft* transcript (Figure 1A). The absence of mature *miR-263a* in these flies suggests that *miR-263a* is the functional product of the *bereft* locus.

*bereft* mutants show defects in the formation of a variety of external sense organs, including loss of the IOB of the eye [11]. In *miR-263a* homozygous mutants and in *Δ263a/bereft^24^* flies ∼80% of IOB were missing (Figure 1C, 1D). The Drosophila genome encodes a second miRNA closely related in sequence to *miR-263a* (Figure S2A). We generated a *miR-263b* deletion allele (*Δ263b*) by homologous recombination and confirmed that mature *miR-263b* was absent in the mutant (Figure S2B). IOB numbers were only modestly reduced in flies lacking *miR-263b* alone (*Δ263b/Df(3L)X-21.2*; Figures S2C, 1D). However, we observed a significant increase in the loss of IOB in flies lacking both *miR-263a* and *miR-263b* compared to *miR-263a* alone (Figures S2C, 1D). These observations suggest that both the *miR-263a* and *miR-263b* miRNAs contribute to IOB formation, with *miR-263a* playing the major role.

In addition to the IOB phenotype, the *miR-263a* and *miR-263b* mutants exhibit other milder defects. The number of large mechanosensory bristles (macrochaetae) on the head and thorax was reduced in *miR-263a* mutant flies compared to controls (Figure S3). Although the magnitude of the reduction in bristle number was small, the difference was statistically significant. There was no significant enhancement of this phenotype in the *miR-263a miR-263b* double mutant (Figure S3). In addition, the *miR-263a* mutant showed reduced viability compared to control flies. Although *miR-263b* showed little effect alone, the *miR-263a miR-263b* double mutant showed a stronger viability phenotype (Figure S4). Our further analysis focused on the bristle phenotypes.

**Figure 2:**
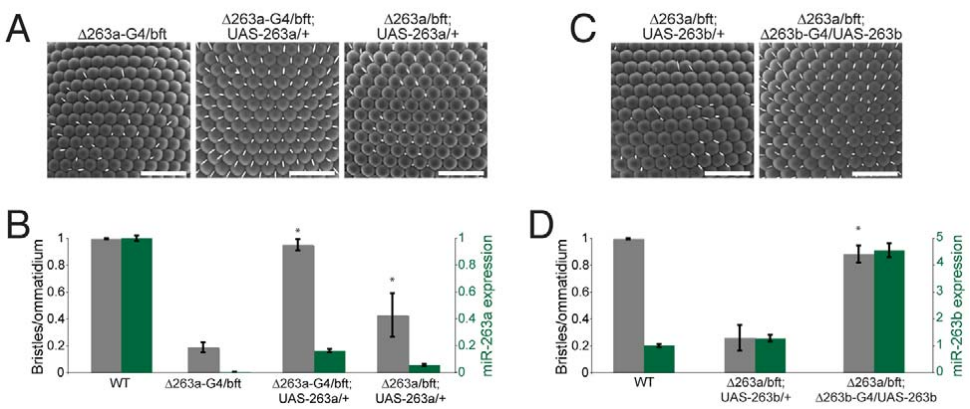
The miR-263a phenotype is rescued by expression of a *miR-263a* or overexpression of a *miR-263b* transgene. (A) SEM of adult eyes from *miR-263a* mutants and *miR-263a* mutants expressing the *UAS-miR-263a* transgene. Δ263a denotes the targeted *miR-263a* deletion, Δ263a-G4 denotes the *miR-263a-Gal4* knock-in allele, UAS-263a denotes the *UAS-miR-263a* transgene, and bft is *bft^24^*. Scale bars = 50 µm. (B) Quantification of IOB numbers (grey bars, left scale) and normalized *miR-263a* miRNA levels measured by miRNA qRT-PCR (green bars, scale at right). Genotypes as indicated in (A), and a wild-type control for comparison. Error bars represent mean ± SD. *N* = 20–25 flies per genotype. [*] *p*<0.001, Student's *t* test comparing to the *miR-263a* mutant. (C) SEM of adult eyes from *miR-263a* mutant flies expressing a *UAS-miR-263b* transgene under the control of *miR-263b-Gal4* (right) or without a Gal4 driver (left). (D) Quantification of IOB numbers and *miR-263b* miRNA levels. Error bars represent mean ± SD. *N* = 20–30 flies per genotype. [*] *p*<0.001, Student's *t* test comparing to *Δ263a/bft; UAS-263b/+*.

In order to verify that the bristle phenotypes are due to loss of the miRNAs, we performed genetic rescue experiments. To this end, we produced Gal4 “knock-in” alleles of *miR-263a* and *miR-263b*, in which the miRNA hairpin sequences were replaced by *Gal4* and *mini-white* (using a modified targeting vector; [13]). Flies carrying the *miR-263a-Gal4* allele in trans to *bereft^24^* or *Δ263a* displayed IOB loss (Figure 2A, 2B; unpublished data). Restoring *miR-263a* expression under the control of *miR-263a-Gal4* using a *UAS-miR-263a* transgene fully suppressed the loss of IOB in *miR-263a* mutant flies (Figure 2A, 2B). Measurement of mature *miR-263a* by quantitative PCR showed that less than 20% of the normal expression level was sufficient to achieve a full rescue (Figure 2B, green bars). A lower level of Gal4-independent expression that results from leakiness of the *UAS-miR-263a* transgene also conferred partial rescue of IOB loss in the *Δ263a/bft* background (Figure 2A, 2B). The bristle loss on head and thorax observed in *miR-263a* mutants was also rescued by Gal4-dependent expression of *UAS-miR-263a* (Figure S3). These data confirm that absence of *miR-263a* is responsible for the loss of mechanosensory bristles observed in *bereft* mutants.

Residues 2–8 at the 5′ end of a miRNA, known as the seed region, are thought to play an important role in miRNA target recognition [10],[14]. *miR-263a* and *miR-263b* differ in sequence, with the seed region being shifted by one residue (Figure S2A). Thus it might be expected that they would have different target spectra. Nonetheless, expression of *UAS-miR-263b* under *miR-263b-Gal4* control was able to rescue the *miR-263a* mutant phenotype (Figure 2C, 2D). Rescue occurred at levels of *miR-263b* several-fold above normal (Figure 2D, green bars), suggesting that *miR-263b* can replace *miR-263a* when over-expressed. In light of the observation that loss of *miR-263b* has a milder impact than loss of *miR-263a*, these results imply that the two miRNAs have targets in common in their role during IOB development.

### Loss of IOB by Apoptosis in *miR-263a* Mutants

Because of the greater dependence of IOB development on *miR-263a*, we focused on the *miR-263a* mutant for more in-depth analysis. Mechanosensory organs are composed of four cells produced by two rounds of asymmetric division of a SOP: the sensory bristle (called the shaft cell), its socket cell, a neuron, and its sheath cell [15]. In *bereft* mutants, all four of these cells are present and properly specified upon completion of IOB cell fate determination [11]. Therefore, *miR-263a* must act at a later stage, after the asymmetric division of the SOP.

**Figure 3:**
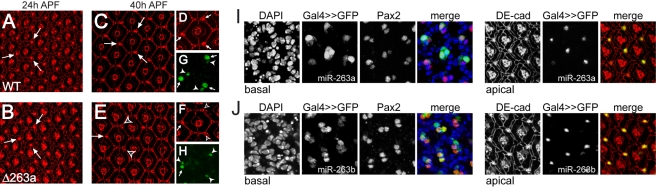
miR-263a inhibits apoptosis in the shaft cells. (A–H) Projections of consecutive confocal sections of segments of pupal retinas. (A, C, D, G) Wild-type controls; (B, E, F, H) *miR-263a* mutants. (A, B) 24 h pupae; (C–H) 40 h pupae. (A–F) Anti-DE-cadherin staining (red). IOB are visible as large brightly labelled cells at alternating vertices of the ommatidial array (arrows). Open arrowheads in (E, F) indicate interommatidial vertices with missing shaft cells. (G, H) Pax2 (green) labels the nuclei of sheath cells (arrowheads) and shaft cells (arrows). The bristle cells have undergone several rounds of endoreplication and so have larger nuclei. The nuclei in (G, H) are located at different focal planes than the cell junctions in (D, F). (I, J) Projections of confocal sections of segments of pupal retinas at 30 h APF. Basal focal planes, left to right: DAPI labelled nuclei (blue in merged image); GFP whose expression was driven with *miR-263a-Gal4* (I) or *miR-263b-Gal4* (J) (green in merged image); Pax2 labelled IOB sheath (small nuclei) and bristle shaft cells (large nuclei; red in merged image). Apical focal planes of the same cells, left to right: cell outlines and IOB labelled with anti-DE-cadherin (red in merged image); *miR-263a* (I) and *miR-263b* (J) expressing cells visualized by GFP expression (green in merged image). Cell junctions (apical) are located at different focal planes than cell nuclei (basal).

To determine when bristle development fails, we examined pupal retinas using an antibody to the cell junction protein DE-cadherin [16]. At 24 h after puparium formation (APF), the hexagonal array of ommatidia is clearly defined; bristle progenitor cells are visible at alternate corners in the hexagonal array of ommatidia (arrows, Figure 3A). At this stage, *miR-263a* mutant retinas were indistinguishable from the controls and bristle shaft progenitor cells were present in normal numbers (Figure 3B).

Approximately one third of the interommatidial cells present at 24 h APF undergo apoptosis during the following 12 h [1],[3]. By 40 h APF, a single row of interommatidial cells surrounds each ommatidium. Bristle shaft progenitor cells appear as brightly labeled cells at three of the six corners (arrows, Figure 3C, 3D). In *miR-263a* mutant retinas, the majority of these cells were missing at 40 h APF (arrowheads, Figure 3E, 3F). Pax2 protein expression marks the nuclei of bristle shaft and sheath cells of external sensory organs (Figure 3G; [17],[18]). The bristle shaft cell grows by an unusual type of cell cycle called endoreplication, in which DNA replication takes place without cell division [19],[20]. These cells have increased ploidy and therefore larger nuclei (arrows, Figure 3G) than the sheath cells (arrowheads). In *miR-263a* mutant retinas many of the larger Pax2 positive nuclei were missing, consistent with bristle shaft cell loss (Figure 3H). We made use of the Gal4 knock-in alleles to direct *UAS-GFP* reporter expression in the endogenous *miR-263a* and *miR-263b* expression domains. Triple labeling to visualize GFP and Pax2, together with DE-cadherin (Figure 3I, 3J), showed that both miRNAs are expressed in the bristle shaft cells during the developmental window in which bristles are lost in the mutants.

**Figure 4:**
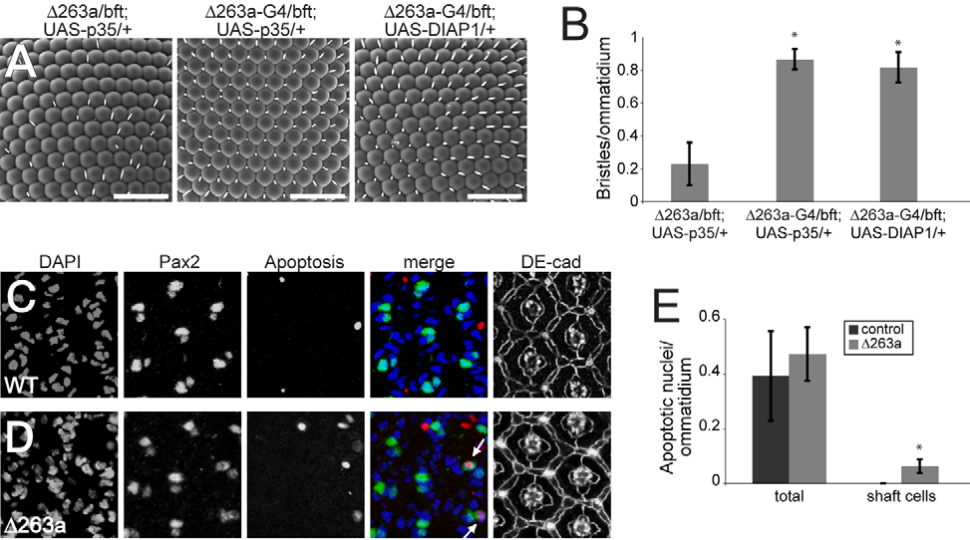
miR-263a inhibits apoptosis in the shaft cells. (A) SEM of adult eyes from *miR-263a* mutant flies carrying *UAS-p35* or *UAS-DIAP1* transgenes. The left panel shows a control in which *UAS-p35* was present but not driven by Gal4. Middle and right panels: transgenes were expressed under *miR-263a-Gal4* control. Scale bars = 50 µm. (B) Quantification of IOB numbers. Genotypes are as indicated in (A). Error bars represent mean ± SD. *N* = 10 flies per genotype. [*] *p*<0.001, Student's *t* test comparing to the control. (C, D) Projections of consecutive confocal sections of segments of pupal retinas at 35 h APF. Left to right: DAPI labelled nuclei (blue in merged image); Pax2 labelled IOB sheath (small nuclei) and bristle shaft cells (large nuclei; green in merged image); Apoptotic nuclei (red in merged image); cell outlines and IOB labelled with anti-DE-cadherin. Arrows in the merged image indicate apoptosis in Pax2-expressing bristle cells. Cell junctions (DE-cad) are located at different focal planes than the nuclei in the merged image. (E) Quantification of apoptotic signal in 35 h APF retinas. Total apoptotic nuclei as well as apoptotic shaft cell nuclei were counted in control flies and *miR-263a* mutants and normalized to the total number of ommatidia analyzed. Error bars represent mean ± SD. *N* = 10 retinas per genotype. [*] *p*<0.001, Student's *t* test compared to the control.

The loss of bristle shaft cells by 40 h APF raised the possibility that they might be eliminated during the normal wave of apoptotic pruning of interommatidial cells. To test this possibility, we made use of *miR-263a*-*Gal4* to express the anti-apoptotic protein p35 in *miR-263a* expressing cells. p35 has been shown to be effective as an inhibitor of apoptosis in Drosophila [21],[22]. Expression of *UAS-p35* using *miR-263a-Gal4* suppressed IOB loss in *miR-263a* mutant flies (Figure 4A, 4B). Similarly, over-expression of the anti-apoptotic protein DIAP1, a direct target of the proapoptotic protein Hid [23], was able to prevent IOB loss in *miR-263a* flies (Figure 4A, 4B). We also monitored programmed cell death in the pupal retina at 35 h APF by visualizing double strand DNA breaks caused by apoptotic endonucleases. In control retinas, we did not observe apoptosis of the Pax2-expressing bristle shaft or sheath cells (Figure 4C). However, apoptotic Pax2-expressing nuclei corresponding to bristle shaft cells were seen in *miR-263a* mutants (arrows, Figure 4D). The total number of apoptotic cells/ommatidium was not significantly elevated in the mutant (Figure 4E), but there was a statistically significant increase in the number of apoptotic cells that were Pax2-expressing bristle shaft cells (*p*<0.001). These findings indicate that *miR-263a* acts to protect these sense organs from the wave of programmed cell death that sweeps over the retina during early pupal development.

### Identification of *miR-263a* Targets

*miR-263a* has several hundred computationally predicted targets [24],[25]. Among these are genes involved in cell proliferation and cell death and a broad range of other biological processes. Endogenous targets are often upregulated in miRNA mutants. Therefore over-expression of a biologically important target in *miR-263a* expressing cells would be expected to result in IOB loss, phenocopying the miR-263a mutant phenotype. *miR-263b-Gal4* was used to drive over-expression of target genes in this series of experiments because it has higher Gal4 activity than *miR-263a-Gal4*. We selected 56 predicted targets for analysis (Table S1). Only two of the candidates caused bristle loss when expressed under control of *miR-263b-Gal4*: *Cyclin E* and *head involution defective (hid)*.

Cyclin E is an essential cell cycle regulator, required for normal cell proliferation and for endoreplication [26]. Endoreplication plays an important role in the growth of bristle shaft and socket cells [19]. Over-expression of *Cyclin E* has been shown to interfere with endoreplication [27],[28] and can suppress bristle shaft cell growth [29]. If *Cyclin E* over-expression is the cause of the bristle loss in *miR-263a* mutants, limiting their capacity to express *Cyclin E* should suppress this phenotype. Bristle loss occurs between 24 and 40 h APF in the mutants. RNA was prepared from pupae at 30 h APF, because the majority of the bristles are not yet lost at this stage. *Cyclin E* mRNA levels were elevated by ∼2.5-fold in RNA samples from *miR-263a* mutants (Figure S5). Removing one copy of the *Cyclin E* gene restored the mRNA to near normal levels but did not rescue the IOB loss phenotype (Figure S5). Although *Cyclin E* is upregulated in the *miR-263a* mutant, this does not appear to contribute to the bristle loss phenotype.

**Figure 5:**
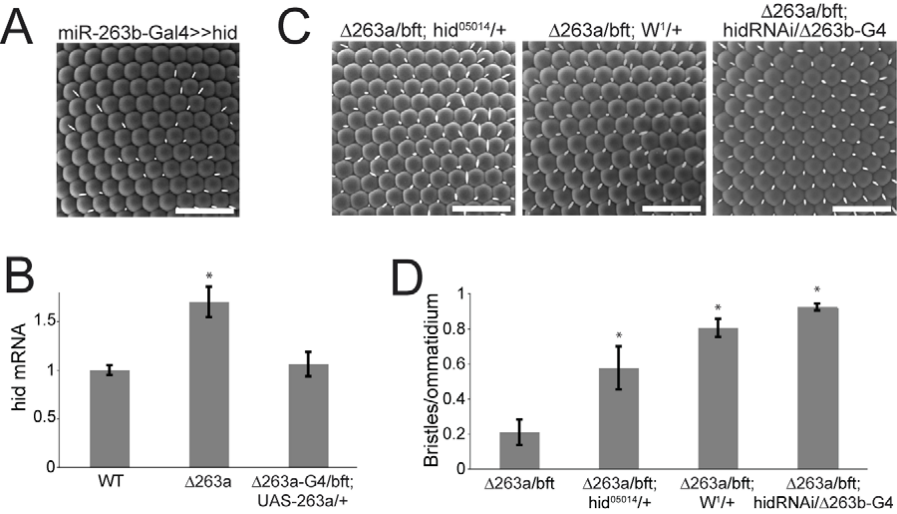
*hid* downregulation by miR-263a is required for IOB formation. (A) SEM of an adult eye expressing the endogenous *hid* gene from the EP line *P[XP]d10274* under *miR-263b-Gal4* control. (B) Normalized *hid* mRNA levels measured by qRT-PCR. RNA was extracted from pupal eye discs of the indicated genotypes at 30 h APF (before the IOB cells are lost in the mutant). Error bars represent mean ± SD of three independent biological replicates for each genotype. [*] *p*<0.001, Student's *t* test compared to the control (WT). (C) SEM of adult eyes from *miR-263a* mutant flies with reduced Hid activity. Left panel: *miR-263a* mutant with one copy of *hid^05014^*; middle panel: *miR-263a* mutant with one copy of the antimorphic *hid* allele *W^1^*; right panel: *miR-263a* mutant expressing a *UAS-hid-RNAi* transgene under control of *miR-263b-Gal4*. Scale bars = 50 µm. (D) Quantification of IOB numbers from flies of the genotypes shown in (C), and *miR-263a* mutant for comparison. Error bars represent mean ± SD for *N* = 20–25 flies per genotype. [*] *p*<0.001, Student's *t* test comparing to the *miR-263a* mutant.

The other candidate, *hid*, encodes an inducer of cell death in Drosophila. *hid* has been shown to play a role in the late stage cell death pathway in the retina [22]. *hid* expression under *miR-263b-Gal4* control caused loss of IOB (Figure 5A). To determine whether *hid* might be a biologically relevant target of *miR-263a* in vivo, we compared *hid* mRNA levels in RNA samples from mutant and control pupal eye discs. *hid* mRNA was 1.6-fold higher in the mutants (Figure 5B). This difference was abolished in *miR-263a* mutant flies rescued by expression of *UAS-miR-263a* under *miR-263a-Gal4* control.

To test whether *hid* over-expression is the cause of bristle loss, we reduced *hid* activity in the *miR-263a* mutant background by introducing the *hid^05014^* loss of function allele [22]. This genetic combination restored IOB numbers to ∼60% of normal levels (Figure 5C, 5D). To further reduce *hid* activity we made use of the *W^1^* allele, which expresses an antimorphic form of *hid* [30], and found a further restoration of IOB number (Figure 5C, 5D). Similarly, reducing *hid* levels by expression of a *UAS-hid-RNAi* transgene under the control of *miR-263b-Gal4* produced a strong suppression of the *miR-263a* mutant phenotype (Figure 5C, 5D). Taken together, these data suggest that *miR-263a* serves to prevent apoptosis in the IOB precursors by limiting *hid* expression during the wave of interommatidial cell pruning.

### *hid* Is a Direct Target of *miR-263a/b*

**Figure 6:**
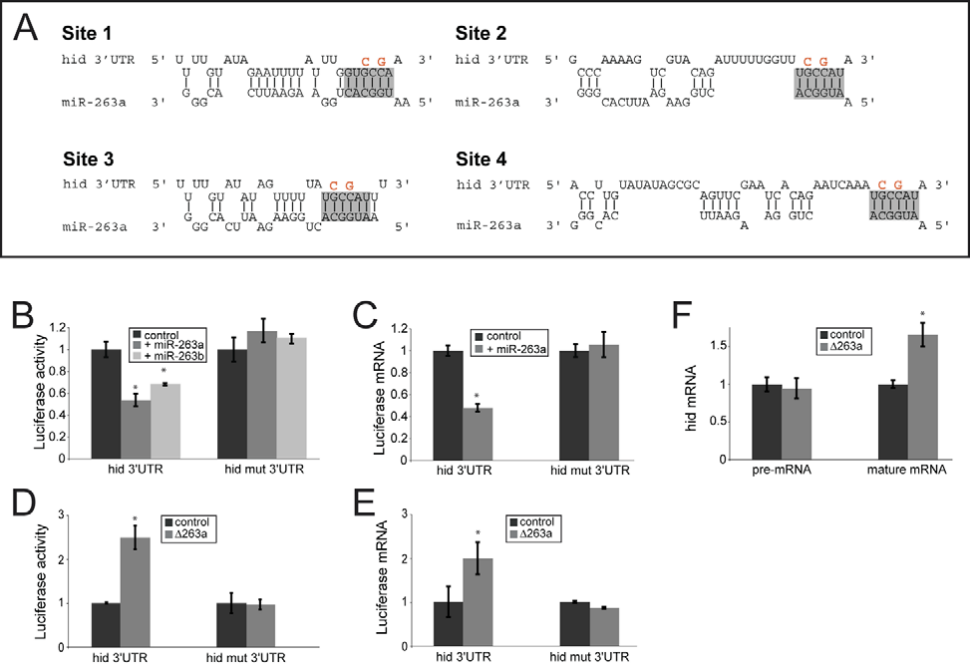
*miR-263a* acts on binding sites in the *hid* 3′UTR. (A) Predicted *miR-263a* target sites in the *hid* 3′UTR. Pairing to the miRNA seed sequence is shaded in grey. Nucleotides changed to generate the target site mutant UTR are shown in red. (B) Normalized firefly luciferase activity from S2 cells transfected to express control and mutated *hid* 3′UTR transgenes. Cells were cotransfected to express *miR-263a* or *miR-263b* or with a vector-only control, and with a plasmid expressing Renilla luciferase as a transfection control. (C) Normalized luciferase mRNA levels measured by qRT-PCR on RNA samples from cells transfected as in (B). (B, C) Error bars: SD based on six independent biological replicates for *miR-263a* and three independent biological replicates for *miR-263b*. (D) Normalized luciferase activity. Cell lysates were obtained from 30 h pupal eye imaginal discs from flies expressing a luciferase reporter carrying the *hid* 3′UTR, or the mutated version of it, in a *miR-263a* mutant or wild-type background. (E) Normalized luciferase mRNA levels. RNA was extracted from samples as in (D). (F) *hid* RNA levels measured using intron-specific primers (left) and exon-specific primers (right). RNA was extracted from pupal eye imaginal discs from control and *miR-263a* mutants at 30 h APF. Unless otherwise indicated, error bars: SD of three independent experiments. [*] *p*<0.001, Student's *t* test comparing to the control levels.

The *hid* 3′UTR contains four potential *miR-263a* binding sites (Figure 6A). To address whether *hid* is a direct target of *miR-263a*, we generated luciferase reporter constructs carrying the full length endogenous *hid* 3′UTR or mutant versions in which two nucleotides of each predicted *miR-263a* site were mutated to compromise pairing to the miRNA seed region (Figure 6A, in red). In S2 cells, co-expression of the luciferase reporter carrying the intact sites with *miR-263a* significantly reduced luciferase activity (Figure 6B, *p*<0.001). This was attributable to reduced luciferase mRNA levels (Figure 6C). These effects were not observed in cells expressing the mutant form of the *hid* reporter (Figure 6B, 6C). We also analyzed the effect of *miR-263b* on the 3′UTR of *hid*. Although *miR-263b* differs from *miR-263a* by three residues, including position 1 of the seed region, *hid* is also a predicted target of *miR-263b* (Figure S6; [24],[25]). Coexpression of *miR-263b* also significantly reduced luciferase activity from the reporter carrying the intact *hid* 3′UTR but not from the reporter in which the miRNA sites were mutated (Figure 6B). Therefore, *miR-263b* and *miR-263a* can each act directly via these sites to regulate *hid* mRNA levels. Differences in the quality of the sites for the two miRNAs may contribute to the apparent difference in their relative potency observed in vivo.

To further assess the functionality of these sites in vivo, we generated transgenic flies expressing the two *hid* 3′UTR luciferase reporters. Luciferase activity levels were compared in pupal retinas dissected from control animals and *miR-263a* mutants. There was no difference in luciferase activity for the transgene carrying the mutant form of the *hid* reporter, but the reporter with the intact sites clearly showed increased luciferase activity in the *miR-263a* mutant (Figure 6D). A similar increase was observed in the level of luciferase mRNA from the *hid* reporter with the intact sites, but not from the reporter with the mutated sites (Figure 6E). Comparable results were obtained comparing GFP reporter transgenes with intact and mutated sites in control and *miR-263a* mutants (Figure S7). We also observed an increase in the level of the endogenous mature *hid* mRNA in the mutant, but not in the level of the *hid* primary transcript, measured by qRT-PCR using intron-specific primers (Figure 6F). Taken together these experiments provide evidence that *miR-263a* acts directly via the sites identified in the 3′UTR to regulate *hid* mRNA levels in vivo. These effects are posttranscriptional, most likely reflecting destabilization of *hid* transcripts.

**Figure 7:**
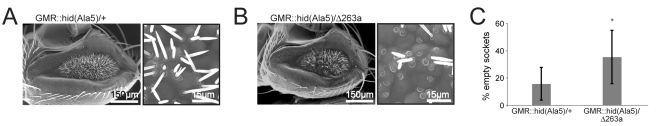
miR-263a limits Hid activity during eye development. (A, B) SEM of adult eyes that express the activated form of *hid* under GMR control. (A) *GMR:hid(Ala5)* alone (B) *GMR:hid(Ala5)* lacking one genomic copy of *miR-263a*. (C) Quantification of missing IOB from flies of the genotypes shown in (A) and (B). The number of IOB socket cells devoid of a shaft cell was normalized to the total number of socket cells. Error bars represent mean ± SD for *N* = 10–15 flies per genotype. [*] *p*<0.005, Student's *t* test comparing to *GMR:hid(Ala5)/+*.

It has been reported previously that excess *hid* activity eliminates photoreceptors and pigment cells, effectively ablating the eye, while sparing the IOB [22],[31]. The result was a tuft of IOB and undifferentiated cuticle in place of the eye. Further increase of *hid* activity led to loss of these cells as well. Based on these observations, it was proposed that IOB might contain high levels of a negative regulator of cell death. To ask whether *miR-263a/b* might be responsible for this activity, we compared the effects of reducing *miR-263a/b* activity in animals expressing the constitutively active form of Hid, *hid(Ala5)*, in the eye. Reducing *miR-263a* levels by removing one copy of the *miR-263a* gene led to fewer IOB, producing a more sparse appearance in the tuft of bristles (Figure 7A, 7B). Because the morphology of the *hid(Ala5)* eyes is strongly perturbed, the effect of reducing the miRNA can be quantified most reliably by counting the number of empty sockets. Every socket should have an IOB hair cell, so the empty socket indicates a specified sense organ lacking the bristle shaft cell. Although the variance of this measure is high, there was a statistically significant increase of ∼2-fold in the proportion of empty sockets (*p*<0.005), indicating loss of IOB due to reduced *miR-263a* activity. Thus reduced activity of the miRNA enhanced the GMR:hid(Ala5) phenotype, suggesting that the miRNA has protective activity even in these extreme conditions of hid over-expression.

Finally, we asked whether the loss of the other mechanosensory bristles in *miR-263a* mutant flies was also a consequence of apoptosis due to elevated Hid activity. To address this we introduced the antimorphic allele of *hid*, *W^1^*, into *miR-263a* miRNA mutant flies and found that mechanosensory bristle loss on head and thorax was also significantly suppressed (Figure S3; *p*<0.001). These observations suggest that *miR-263a/b* play a protective role, preventing the loss of mechanosensory cells due to *hid*-induced apoptosis.

## Discussion

### microRNAs and Robustness

Robustness of a biological system can be thought of in terms of the mechanisms that ensure stability. In developmental terms, perturbation can come in the form of fluctuating levels of intercellular signaling and/or gene expression and can be intrinsic or of environmental origin. Gene regulatory networks have properties that can help to confer stability by buffering the effects of fluctuations in gene expression (reviewed in [32]). Computational analysis has suggested that miRNAs are over-represented in gene regulatory networks in animals, suggesting that they confer useful regulatory possibilities [33],[34]. However to date few examples have been investigated experimentally in terms of biological processes that confer robustness during development of multicellular organisms. A recent study has provided compelling evidence for a miRNA acting to confer robustness to sensory organ specification in Drosophila [7]. In this report we examine the role of *miR-263a/b* in conferring robustness to sensory organ survival during a developmental pruning process.

*miR-7* was shown to act in two molecularly distinct feed-forward loops required for sense organ specification [7]. One pair of feedforward motifs involve the transcription factors Yan and Pointed, which mediate Notch and EGF signaling to control R8 photoreceptor specification. The second involves the transcription factors E(spl) and Atonal to control SOP specification. In both examples *miR-7* is induced by one of the transcription factors to confer repression on the other. The requirement for *miR-7* activity in these patterning processes is not evident under normal, environmentally stable, conditions. However, it can be revealed under destabilizing conditions, including severe environmental fluctuation, or in sensitized genetic backgrounds. Thus miR-7 acts to provide stability to these molecular networks.

We have explored the possibility that *miR-263a/b* might function in a regulatory feed-forward network to control *hid* both directly and indirectly. The RAS/MAPK pathway has been reported to regulate *hid* activity at two levels. Hid activity is controlled by direct phosphorylation mediated by MAPK [31]. Signalling through the EGF/RAS/MAPK pathway protects cells of the developing eye from apoptosis, through MAPK mediated regulation of Hid activity. In addition MAPK signalling represses *hid* transcription [8]. Intriguingly, an upstream element of the MAPK pathway, *Ras85D*, is a predicted target of *miR-263a*. In this scenario, *miR-263a* would act directly to repress *hid* and indirectly via the RAS/MAPK pathway (illustrated in Figure S8). Negative regulation of *Ras85D* by *miR-263a* would repress MAPK activity and alleviate repression of *hid* transcription and of Hid protein activity. The result is a so-called “incoherent” motif [32], in which the two branches have opposing effects on their shared target. A prediction of this model is that *hid* transcription should decrease in the *miR-263a* mutant due to elevation of MAPK activity. However, as shown in Figure 6F, *hid* primary transcript levels were not significantly affected in the *miR-263a* mutant, although mature *hid* mRNA levels increased due to loss of miRNA direct mediated repression. As a further test, *Ras85D* mRNA levels were measured in pupal eye discs dissected from control animals and *miR-263a* mutants. There was no significant difference (Figure S8). Although the topology of this predicted network suggested a potential role in control of *hid* activity, we do not find sufficient evidence to support the biological relevance of this network in vivo. This example highlights the importance of experimental validation in vivo in assessing such predictions.

Our findings suggest a role for the *miR-263a/b* miRNAs in conferring robustness of a different sort, ensuring the survival of sense organ cells, after they have been specified by the developmental patterning process. In this scenario the fluctuating cellular landscape derives from triggering cell death through randomly variable activity of the pro-apoptotic gene *hid.* Under normal conditions, this rarely, if ever, causes bristle loss. However in *miR-263a/b* mutants sporadic bristle loss was seen and was attributable to elevated *hid* activity. As in the case of the macrochaetae, loss of individual interommatidial cells is a stochastic process. In each of the single mutants we observed a variable loss of IOB, suggesting that the chance of any given nascent IOB cell succumbing to apoptosis has increased in the absence of the protective effect of *miR-263a/b*. The overall robustness of the pruning process is impaired.

In the *miR-7* case, the mutants do not show any defect under normal conditions, but the limits to the robustness of the system can be revealed by environmental perturbation. This is consistent with a scenario in which robustness derives from a gene regulatory network designed to buffer molecular perturbation. Based on the observations of Li et al. [7], we examined whether the severity of the *miR-263a/b* mutant phenotype would be affected by environmental fluctuation to increase noise but found no effect (unpublished data). We also did not find evidence that *miR-263a/b* act in the context of a gene regulatory network. Instead, *miR-263a/b* appears to function in a different context, acting as a buffer in a biological process that is inherently stochastic. In this way miRNA activity is used to ensure that apoptotic cell death is not allowed to compromise specific cells. It is noteworthy that as little as 20% of normal *miR-263a* levels are sufficient to support IOB development. This implies a considerable buffering capacity to ensure that the process of IOB formation is robust in a fluctuating developmental landscape.

### microRNAs and Apoptosis

Several other microRNAs have been implicated in the control of apoptosis in Drosophila. *bantam* miRNA functions during tissue growth and regulates *hid*, to prevent proliferation induced apoptosis [9]. However, under normal conditions *bantam* regulation of *hid* does not appear to impact upon apoptotic pruning or on survival of sense organs (our unpublished observation). *miR-14* has also been reported to be anti-apoptotic [35]. *miR-14* mutants do not impact on IOB apoptosis ([36] and our unpublished observations). Similarly, *miR-8* mutants show apoptosis in the CNS [37] but do not have an IOB phenoytpe. Finally, members of the *miR-2* family of miRNAs have been shown to regulate the propaptotic genes, *reaper*, *grim*, and *sickle* in S2 cell over-expression assays or in reporter transgene assays in vivo [10],[38]. Injection of antisense oligonucleotides to deplete members of this family have been reported to cause apoptosis in the embryo [39], but none of the mutants that affect the members of this family have yet shown any role in apoptotic pruning ([40] and our unpublished observations). We do not exclude the possibility that potential phenotypes might be masked by functional redundancy among family members. The available evidence suggests that *miR-263a/b* may have a dedicated role in controlling *hid*-induced apoptosis during developmental pruning of interommatidial cells.

### microRNA Targets

Most miRNAs are predicted computationally to have many possible targets. Yet our survey of over 50 candidates yielded only one target, *hid*, for which we have functional evidence in vivo. It may be of interest in this context to consider data from analysis of Drosophila miRNA target predictions. A high proportion of predicted targets are regulated in cell-based miRNA over-expression assays (sample refs: [5],[41]–[44]). This provides evidence that the miRNA, when present abundantly, can regulate the predicted target site. There are fewer examples in which over-expression of a predicted target can be shown to contribute directly to causing a specific miRNA mutant phenotype in vivo. In most such cases only one or a few targets have been implicated (reviewed in [45],[46]; see also [47]–[49]). For miRNA mutants that have been studied in detail, evidence has begun to emerge that different aspects of the mutant phenotype may result from misregulation of different targets in different tissues. *miR-8* is a good example, with well characterized phenotypes linked to different targets in three different tissues: Atrophin for neurodegeneration in the CNS [37], Enabled for neuromuscular junction development [48], and U-shaped in the adipose tissue to control tissue growth [49]. Finding a few different key targets in different tissues may prove to be a common theme.

The genetic evidence presented here identifies *hid* as a key target of the *miR-263* family in supporting bristle development. A priori we cannot exclude that there might be other important targets. But we note that only *hid*, of the 56 candidates tested, fulfilled two essential criteria: (1) being able to mimic the miRNA mutant phenotype when over-expressed in the miRNA expressing cells and (2) being able to suppress the miRNA mutant phenotype when its level was reduced in the miRNA expressing cells. Further investigation of the *miR-263* family miRNA mutants may lead to identification of targets important for other aspects of the miRNA function, such as the reduced viability observed in the double mutants.

### A Conserved Family of microRNAs Implicated in Maintenance of Sense Organ Survival

Drosophila *miR-263a* and *miR-263b* are members of a conserved family of miRNAs, including mammalian *miR-183*, *miR-96* and *miR-182*, and *miR-228* in C. elegans. Interestingly, members of this family display conservation of expression in ciliated sensory organs in vertebrate and invertebrate organisms [50]. *miR-183*, *miR-96*, and *miR-182* are expressed in sensory hair cells in mammalian auditory and vestibular organs, as well as in sensory cells of the eye and ear in zebrafish and chicken [51]–[55]. *C. elegans miR-228* is expressed in chemosensory and mechanosensory sensilla [50]. Drosophila *miR-263a* and *miR-263b* are expressed in sense organ precursors in embryos [11],[56] and in mechanosensory organs of the eye, antenna, and haltere ([11],[50], this report). The high degree of sequence conservation and expression in sensory organs across phyla raises the possibility that a common ancestor of these miRNAs was associated with sensory cell development and function [50].

Further support for the idea of conservation of function comes from the observation that mutations affecting *miR-96* have been identified as the cause of auditory hair cell degeneration and non-syndromic progressive hearing loss in mice and humans [57],[58]. Depletion of all miRNAs using conditional *dicer* mutants in the mouse also leads to defects in inner ear hair cell development [59],[60]. Whether there is more than a coincidental similarity to the role of *miR-263a/b* in support of sensory hair development in Drosophila remains to be determined. Superficially the way in which these sense organs are lost appears to differ. In the fly, the mechanosensory cells are lost due to apoptosis in the *miR-263a/b* mutants. In the mammalian systems, mature differentiated sensory cells appear to be lost through degeneration. In the case of the *miR-96* mutant this could be due to inappropriate regulation of genes that are not normally *miR-96* targets due to the change in sequence of the mutant miRNA seed, but in the case of dicer conditional mutants it is more likely due to loss of normal miRNA mediated target regulation. Whether this involves apoptosis is not known. Intriguingly, there is evidence suggesting an anti-apoptotic role for *miR-182* and related family members in human cancers [61],[62]. So the possibility of an underlying conservation of mechanism exists.

## Methods

### Plasmids and Fly Strains

Canton-S flies were used as the wild-type control. EP lines were obtained from the Bloomington, Szeged, and Exelixis stock centres. The *hid* UAS-RNAi strain was obtained from the VDRC. *bft^24^*, *bft^225^*, and *bft^6B^* were provided by Rolf Bodmer. *hid^05014^* and GMR:hid(Ala5) were provided by Hermann Steller. *W^1^*, Df(3L)X-21.2, UAS-p35, and UAS-Diap1 were obtained from the Bloomington stock centre. UAS-miR-263a and UAS-miR-263b lines were made by cloning a 300 base pair genomic fragment containing the miRNA hairpin into the 3′UTR of dsRed in pUAST, as described in [10]. The GFP and luciferase *hid* 3′UTR reporters were made by cloning the 2.2 kb hid 3′UTR after GFP or luciferase, under control of the tubulin promoter [5],[9]. *hid* UTR reporters with mutated miR-263a/b sites were generated by PCR using primers designed to change the seed region from TGCCA into TCCGA. PCR products were sequence verified.

### Mutant Generation

Ends-out homologous recombination was performed as described [12]. As *miR-263b* is located in an intron of *CG32150*, we removed the intron-containing mini-white gene cassette and confirmed that splicing of *CG32150* mRNA was not affected in the *ΔmiR-263b* mutants by comparing the level of spliced mRNA using qRT-PCR. *miR-263a* and *miR-263b* Gal4 knock-in alleles were made using a modified targeting vector [13].

### Cell Transfection and Luciferase Assays

Luciferase reporters and miRNA expression plasmids were expressed under the control of the tubulin promoter. S2 cells were transfected in 6-well plates with 1,000 ng of miRNA expression plasmid or empty vector, 500 ng of firefly luciferase reporter plasmid, and 500 ng of Renilla luciferase DNA as a transfection control. Transfections were performed with triplicate technical replicates in at least three independent experiments. At 60 h post-transfection, dual luciferase assays (Promega) were performed on a portion of the transfected cells. The other portion was pelleted and dissolved in Trizol reagent (Invitrogen) for total RNA extraction. For luciferase assays on pupal retinas, retinal tissue was dissected and immediately lysed in passive lysis buffer (Promega). Luciferase activity was normalized to total protein content, measured on the same sample using the Bradford method (Bio-Rad).

### RNA Analysis

Northern blots on small RNA were carried out according to [63]. 5 µg of total RNA extracted from adult flies were loaded per lane. The blot was probed with an oligonucleotide complementary to miR-263a, 5′end-labeled with [32]-P. For miRNA qRT-PCR, primer sets designed to amplify mature miR-263a and miR-263b were obtained from Applied Biosystems. Reverse transcription was done on 100 ng of total RNA. miRNA levels were calculated relative to miR-8, after having confirmed that miR-8 levels remain constant in the relevant fly strains. For mRNA qRT-PCR, total RNA was treated with RNase-free DNase (Promega) to eliminate DNA contamination. First strand synthesis used random hexamer primers and SuperScript RT-III (Invitrogen). Samples were RNaseH-treated after the RT reaction and used for qRT-PCR. Measurements were normalized to mitochondrial large ribosomal RNA (mtlrRNA1, AAAAAGATTGCGACCTCGAT and AAACCAACCTGGCTTACACC) or to the transfection control Renilla luciferase mRNA (CGGACCCAGGATTCTTTT and TTGCGAAAAATGAAGACCT). Primers for *hid* pre-mRNA: TGAAGGTGTTCTCCGATTCC and ATCTCACCCAGCGCTCTTTA. Primers for mature *hid* mRNA: GAGAACGACAAAAGGCGAAG and CAAAACGAAAACGGTCACAA. Firefly luciferase primers: CCGCCGTTGTTGTTTTG and CTCCGCGCAACTTTTTC. GFP primers: GCAGTGCTTCAGCCGCTA and AGCCTTCGGGCATGGC.

### Scanning Electron Microscopy and IOB Counts

Adult flies were fixed overnight in 2.5% glutaraldehyde at 4°C, washed 3×15 min with PBS, dehydrated in a series of ascending ethanol concentrations, critical point dried, mounted on stubs, and coated with gold. Eyes were imaged with a JSM-6360LV scanning microscope (JEOL, Tokyo, Japan). The orientation, contrast, and brightness of the images were adjusted using ImageJ. For the quantification of bristle numbers, a high-resolution image of a whole eye was printed and the maximal visible surface delimited, usually 300–500 ommatidia. The number of visible IOBs was counted and divided by the total number of corners where IOBs would be expected or, for Figure 7, by the total number of bristle sockets. 10–30 eyes were analyzed for each genotype.

### Immunocytochemistry

To stage pupae, white pre-pupae were collected and aged at 25°C until dissection. Pupal eye imaginal discs were dissected and fixed in 4% formaldehyde for 20 min on ice. The following primary antibodies were used: rat anti-Dcad2 1∶40 (Developmental Studies Hybridoma Bank), rabbit anti-DPax2 1∶50 (a gift from Erich Frei and Markus Noll), and chicken anti-GFP 1∶1000 (Abcam). Fluorescent secondary antibodies were from Jackson Laboratories. Samples were mounted in Vectashield (Vector Laboratories). Detection of apoptotic cells in pupal eye discs was done using the Apoptag ISOL dual fluorescence kit (Chemicon). Immunofluorescence images were collected using a Leica SPE confocal microscope and processed using ImageJ. Quantification of apoptotic nuclei was done using z-projections of confocal sections. Total apoptotic nuclei as well as apoptotic shaft cell nuclei were counted and normalized to the total number of ommatidia analyzed.

## Supporting Information

Figure S1 ***miR-263a*** **is absent in** ***bft*** **lines.** Northern blot showing mature *miR-263a* in total RNA extracted from adult control flies (WT) and the three *bft* homozygous mutants described in Hardiman et al. 2002 [11]. A probe for Valine tRNA was used to monitor loading.
(0.47 MB TIF)

Figure S2 ***miR-263b*** **contributes to IOB formation.** (A) Aligned sequences of *miR-263a* and *miR-263b*. The three differing nucleotides are highlighted in red. The seed region (grey shading) comprises nucleotides 2 to 8 of the miRNA. (B) Normalized *miR-263b* levels in adult flies, measured by miRNA qPCR. Δ263b: *miR-263b* knockout allele, Δ263b-G4/Def: *miR-263b-Gal4* knock-in allele in trans with the genomic deficiency *Df(3L)X-21.2*. (C) SEM of adult eyes from *miR-263b* single mutant (two representative examples) and *miR-263a miR-263b* double mutant flies. Scale bars = 50 µm.
(0.45 MB TIF)

Figure S3 **Absence of** ***miR-263*** **causes loss of bristles on head and thorax.** Quantification of macrochaetae on head and thorax of adult flies: wild-type (WT), *miR-263a* mutant (Δ263a/bft), *miR-263a* mutant expressing an *UAS-miR-263a* transgene (rescue flies: Δ263a-G4/bft; UAS-263a/+), *miR-263a miR-263b* double mutant (Δ263a/bft; Δ263b/Def, where Def represents the genomic deficiency *Df(3L)X-21.2*), *miR-263a* mutant with one copy of the antimorphic *hid* allele *W^1^* (Δ263a/bft; W^1^/+). Error bars represent mean ± SD for *N* = 50 flies per genotype. [*] = *p*<0.001, using two-tailed unpaired Student's *t* test comparing to *Δ263a/bft* flies. Macrochaetae numbers in the *miR-263a miR-263b* double mutant differed slightly, but not statistically significantly, from those in *miR-263a* mutants. Single mutant: 95.3%, double mutant 94.4%, *p* = 0.12 using two-tailed unpaired Student's *t* test comparing the single and double mutants.
(0.11 MB TIF)

Figure S4 **Viability of** ***miR-263a*** **and** ***miR-263b*** **mutants.** Viability of different *miR-263a* and *miR-263b* mutant lines. Numbers indicate the percentage of flies observed relative to what is expected if fully viable. Hatched: percentage of embryos that hatched (*n* = 500 embryos counted); pupated: percentage of the resulting first instar larvae that pupated; eclosed: percentage of adult flies that emerged from these pupae. 1-d-old is the percentage of adult flies surviving 1 d after eclosion. For ease of comparison, the numbers in each category for *w^1118^* were set to 100%. *w^1118^* flies were used as a control. Δ263a/bft: *miR-263a* mutant, Δ263b/Def: *miR-263b* mutant, where Def represents the genomic deficiency *Df(3L)X-21.2*, Δ263a/bft; Δ263b/Def: *miR-263a miR-263b* double mutant.
(0.38 MB TIF)

Figure S5 ***CycE*** **over-expression does not cause the IOB phenotype.** (A) Normalized *Cyclin E* mRNA levels in flies with the indicated genotype. RNA was extracted from whole 30 h APF pupae. WT: wild-type; Δ263a/bft: trans-heterozygous *miR-263a* mutant; Δ263a,CycE^AR95^/bft: *miR-263a* mutant carrying one copy of *CycE^AR95^*, a null allele of *CycE*. Bars represent mean ± SD of three independent batches of pupae. (B) SEM of adult eyes from flies with the indicated genotype. Scale bars = 50 µm. *CycE* is elevated in *miR-263a* mutants. Reducing the dosage of *CycE* to wild-type levels does not rescue bristle loss, which indicates that over-expression of *CycE* is not the cause of the miR-263a phenotype.
(0.44 MB TIF)

Figure S6 ***miR-263b*** **target sites in the** ***hid*** **3′UTR.** Predicted *miR-263b* target sites in the *hid* 3′UTR. Pairing to the miRNA seed sequence is shaded in grey. Nucleotides changed to generate the target site mutant UTR are shown in red.
(0.59 MB TIF)

Figure S7 ***miR-263a*** **regulates a GFP transgene carrying the** ***hid*** **3′UTR.** Normalized GFP mRNA levels measured by qRT-PCR. RNA was extracted from 30 h pupal eye imaginal discs from flies expressing a GFP reporter carrying the *hid* 3′UTR or a mutated version of it, in a *miR-263a* mutant or wild-type control background. Bars represent mean ± SD of three independent experiments. [*] *p*<0.001 using two-tailed unpaired Student's *t* test comparing to the control levels.
(0.11 MB TIF)

Figure S8 **A predicted feed-forward regulatory network involving mR-263a, hid, and the MAPK pathway.** (A) Topology of the predicted feed-forward network: Negative regulation of RAS by the miRNA would repress MAPK activity and alleviate repression of hid transcription and of HID protein activity. In other words the effect of the miRNA on the MAPK branch would be to increase hid transcription and Hid protein activity. In the *miR-263a* mutant (illustrated at right), the predicted elevation of MAPK activity should lower *hid* activity, acting in opposition to the increase in *hid* mRNA levels caused by the *miR-263a* mutant. (B) *Ras85D* mRNA levels in pupal eye discs of control and *miR-263a* mutants. *Ras85D* is on the list of predicted *miR-263a* targets (but not on the *miR-263b* list due to differences in the seed sequence). Ras mRNA levels were measured by Q-RT-PCR on RNA from pupal eye discs dissected from control animals and *miR-263a* mutants, as well as rescued mutants. There was no significant difference.
(0.10 MB TIF)

Table S1 **List of tested candidate genes, with the corresponding EP lines and results (IOB loss: yes or no) when expression is driven with** ***miR-263b-Gal4*** **.**
(0.09 MB DOC)

## Abbreviations

APF: (hours) after puparium formation
GFP: green fluorescent protein
*hid*: *head involution defective*
IOB: interommatidial bristle
miRNA: microRNA
ng: nanograms
qRT-PCR: quantitative real-time polymerase chain reaction
RNAi: RNA interference
SOP: sense organ precursor
UAS: (Gal4 dependent) upstream activating sequence
µg: micrograms
UTR: untranslated region
