# Supplementary figures and images for: Drosophila microRNAs 263a/b Confer Robustness during Development by Protecting Nascent Sense Organs from Apoptosis
Source: PLoS Biol. 2010 Jun 15;8(6):e1000396. doi: 10.1371/journal.pbio.1000396 (PMC2885982; doi:10.1371/journal.pbio.1000396)

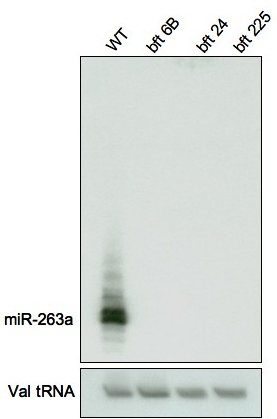

Supplement: Figure S1 — miR-263a is absent in bft lines. Northern blot showing mature miR-263a in total RNA extracted from adult control flies (WT) and the three bft homozygous mutants described in Hardiman et al. 2002 [11]. A probe for Valine tRNA was used to monitor loading. (0.47 MB TIF) [file pbio.1000396.s001.tif]

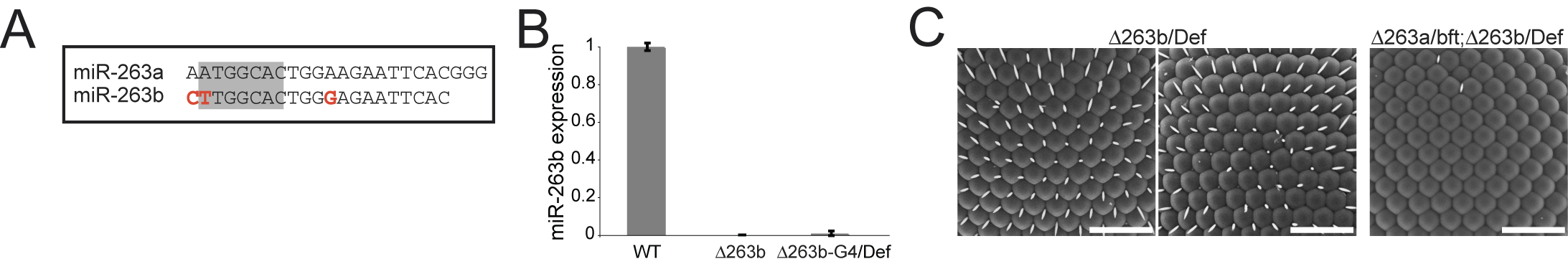

Supplement: Figure S2 — miR-263b contributes to IOB formation. (A) Aligned sequences of miR-263a and miR-263b. The three differing nucleotides are highlighted in red. The seed region (grey shading) comprises nucleotides 2 to 8 of the miRNA. (B) Normalized miR-263b levels in adult flies, measured by miRNA qPCR. Δ263b: miR-263b knockout allele, Δ263b-G4/Def: miR-263b-Gal4 knock-in allele in trans with the genomic deficiency Df(3L)X-21.2. (C) SEM of adult eyes from miR-263b single mutant (two representative examples) and miR-263a miR-263b double mutant flies. Scale bars = 50 µm. (0.45 MB TIF) [file pbio.1000396.s002.tif]

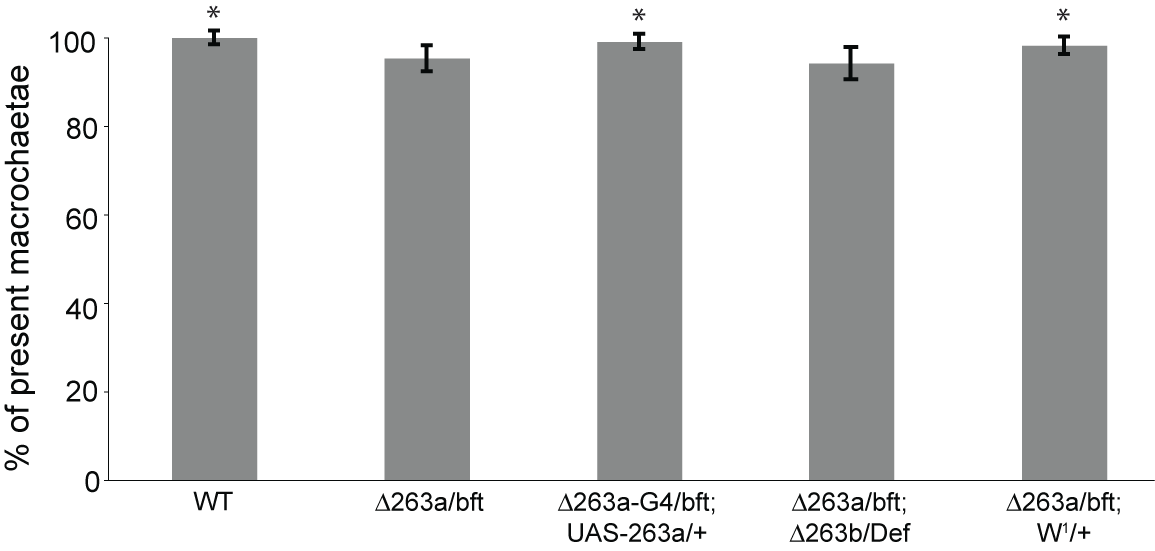

Supplement: Figure S3 — Absence of miR-263 causes loss of bristles on head and thorax. Quantification of macrochaetae on head and thorax of adult flies: wild-type (WT), miR-263a mutant (Δ263a/bft), miR-263a mutant expressing an UAS-miR-263a transgene (rescue flies: Δ263a-G4/bft; UAS-263a/+), miR-263a miR-263b double mutant (Δ263a/bft; Δ263b/Def, where Def represents the genomic deficiency Df(3L)X-21.2), miR-263a mutant with one copy of the antimorphic hid allele W1 (Δ263a/bft; W1/+). Error bars represent mean ± SD for N = 50 flies per genotype. [*] = p<0.001, using two-tailed unpaired Student's t test comparing to Δ263a/bft flies. Macrochaetae numbers in the miR-263a miR-263b double mutant differed slightly, but not statistically significantly, from those in miR-263a mutants. Single mutant: 95.3%, double mutant 94.4%, p = 0.12 using two-tailed unpaired Student's t test comparing the single and double mutants. (0.11 MB TIF) [file pbio.1000396.s003.tif]

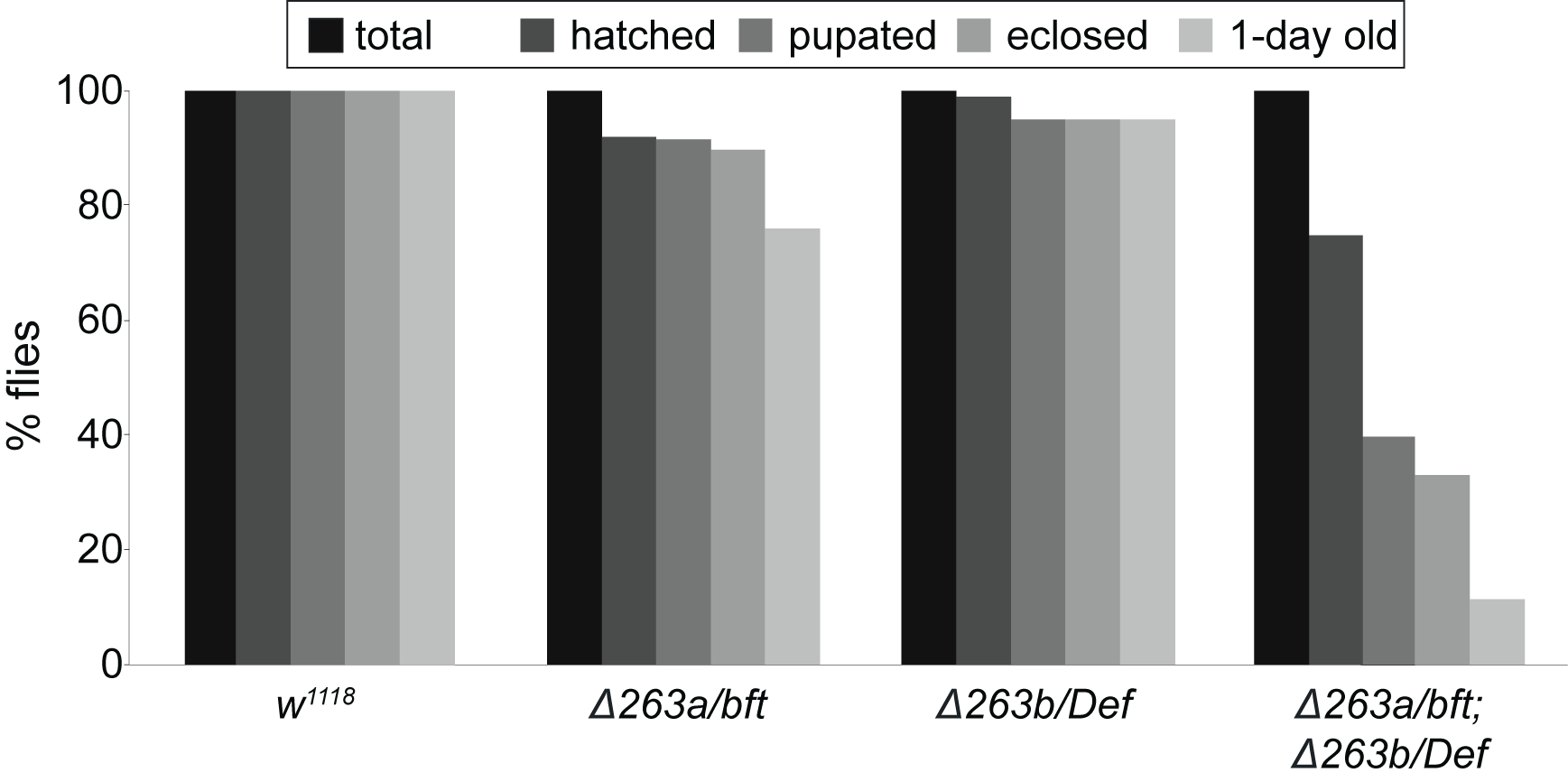

Supplement: Figure S4 — Viability of miR-263a and miR-263b mutants. Viability of different miR-263a and miR-263b mutant lines. Numbers indicate the percentage of flies observed relative to what is expected if fully viable. Hatched: percentage of embryos that hatched (n = 500 embryos counted); pupated: percentage of the resulting first instar larvae that pupated; eclosed: percentage of adult flies that emerged from these pupae. 1-d-old is the percentage of adult flies surviving 1 d after eclosion. For ease of comparison, the numbers in each category for w1118 were set to 100%. w1118 flies were used as a control. Δ263a/bft: miR-263a mutant, Δ263b/Def: miR-263b mutant, where Def represents the genomic deficiency Df(3L)X-21.2, Δ263a/bft; Δ263b/Def: miR-263a miR-263b double mutant. (0.38 MB TIF) [file pbio.1000396.s004.tif]

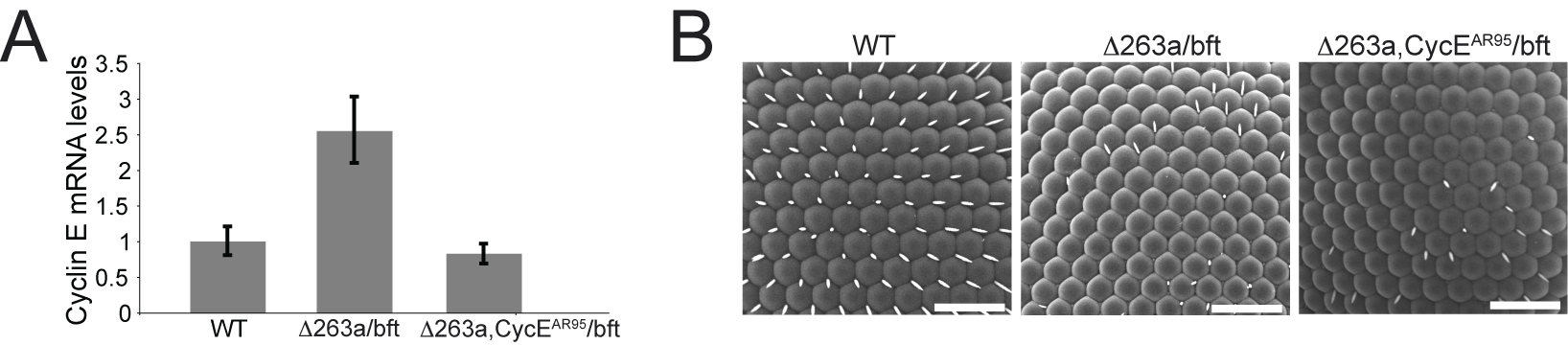

Supplement: Figure S5 — CycE over-expression does not cause the IOB phenotype. (A) Normalized Cyclin E mRNA levels in flies with the indicated genotype. RNA was extracted from whole 30 h APF pupae. WT: wild-type; Δ263a/bft: trans-heterozygous miR-263a mutant; Δ263a,CycEAR95/bft: miR-263a mutant carrying one copy of CycEAR95, a null allele of CycE. Bars represent mean ± SD of three independent batches of pupae. (B) SEM of adult eyes from flies with the indicated genotype. Scale bars = 50 µm. CycE is elevated in miR-263a mutants. Reducing the dosage of CycE to wild-type levels does not rescue bristle loss, which indicates that over-expression of CycE is not the cause of the miR-263a phenotype. (0.44 MB TIF) [file pbio.1000396.s005.tif]

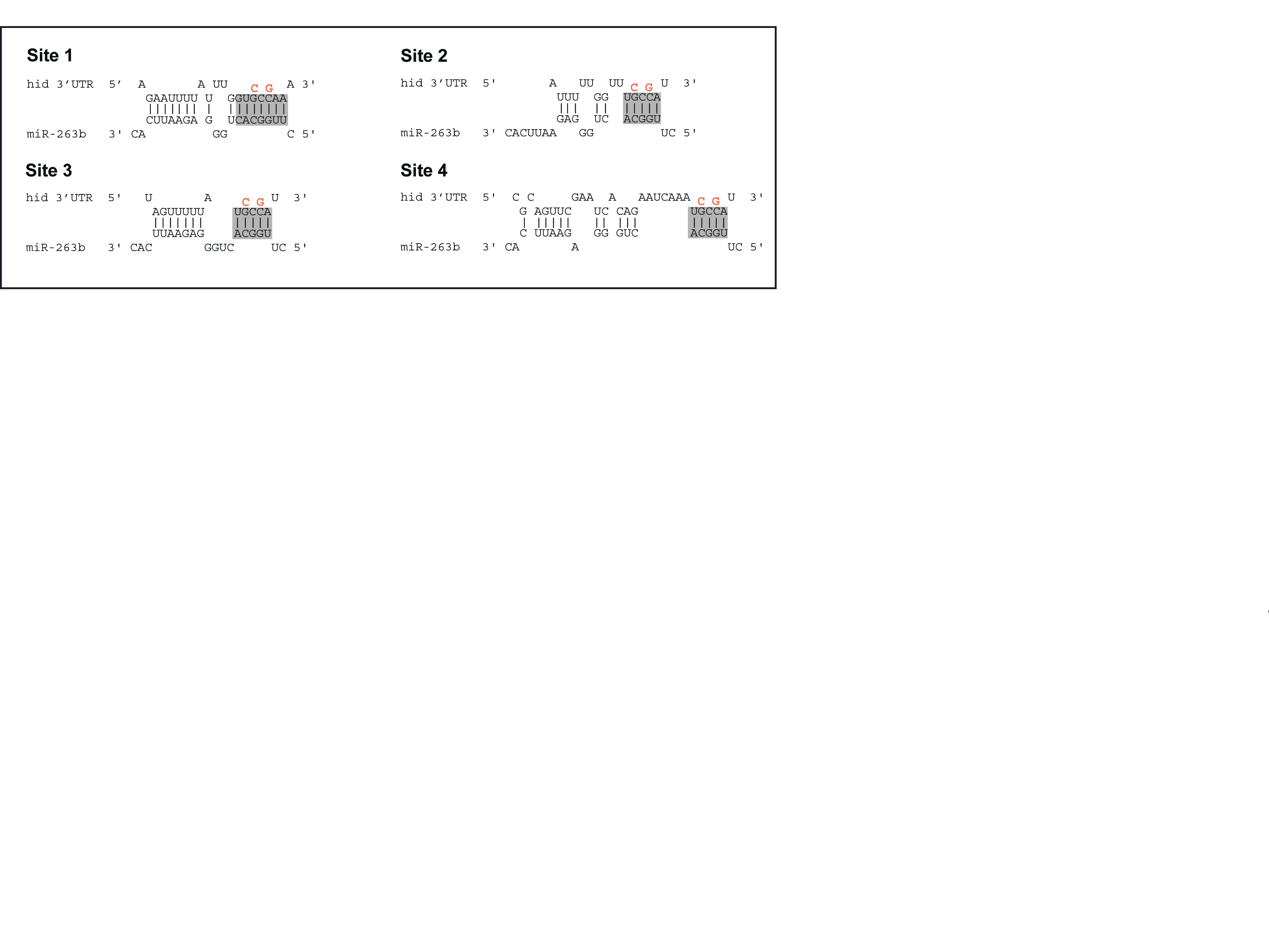

Supplement: Figure S6 — miR-263b target sites in the hid 3′UTR. Predicted miR-263b target sites in the hid 3′UTR. Pairing to the miRNA seed sequence is shaded in grey. Nucleotides changed to generate the target site mutant UTR are shown in red. (0.59 MB TIF) [file pbio.1000396.s006.tif]

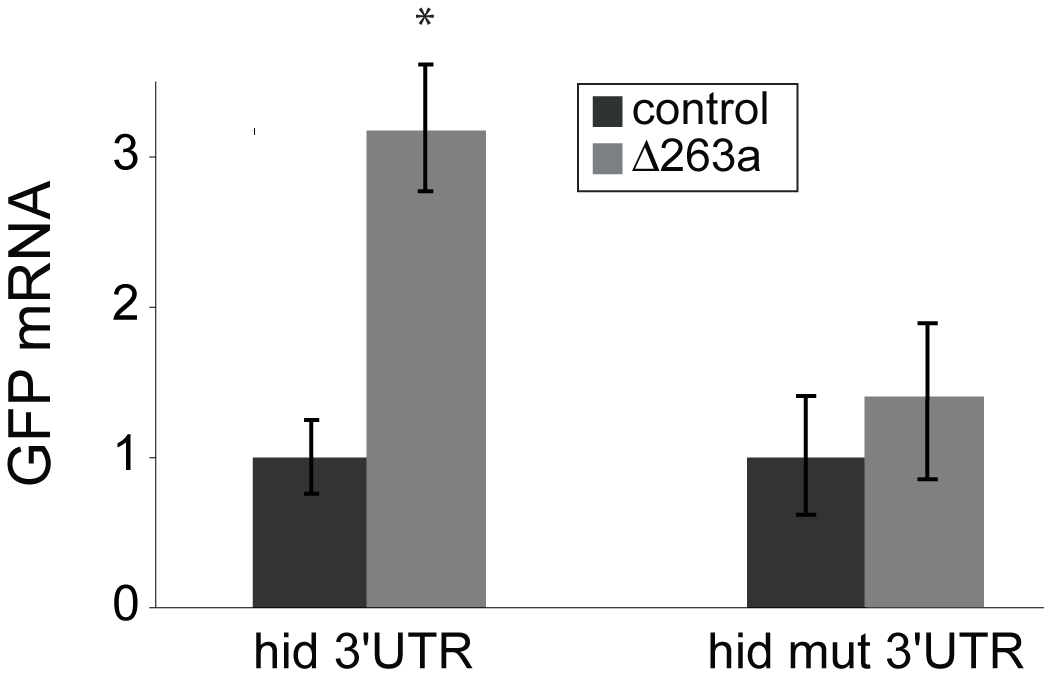

Supplement: Figure S7 — miR-263a regulates a GFP transgene carrying the hid 3′UTR. Normalized GFP mRNA levels measured by qRT-PCR. RNA was extracted from 30 h pupal eye imaginal discs from flies expressing a GFP reporter carrying the hid 3′UTR or a mutated version of it, in a miR-263a mutant or wild-type control background. Bars represent mean ± SD of three independent experiments. [*] p<0.001 using two-tailed unpaired Student's t test comparing to the control levels. (0.11 MB TIF) [file pbio.1000396.s007.tif]

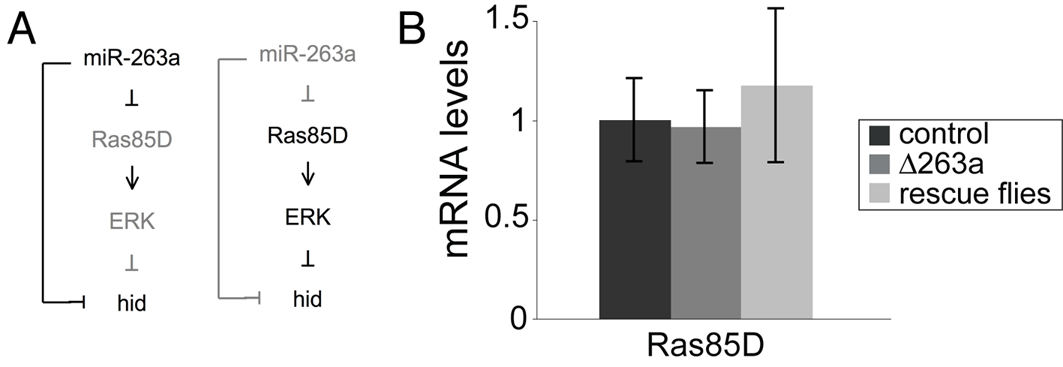

Supplement: Figure S8 — A predicted feed-forward regulatory network involving mR-263a, hid, and the MAPK pathway. (A) Topology of the predicted feed-forward network: Negative regulation of RAS by the miRNA would repress MAPK activity and alleviate repression of hid transcription and of HID protein activity. In other words the effect of the miRNA on the MAPK branch would be to increase hid transcription and Hid protein activity. In the miR-263a mutant (illustrated at right), the predicted elevation of MAPK activity should lower hid activity, acting in opposition to the increase in hid mRNA levels caused by the miR-263a mutant. (B) Ras85D mRNA levels in pupal eye discs of control and miR-263a mutants. Ras85D is on the list of predicted miR-263a targets (but not on the miR-263b list due to differences in the seed sequence). Ras mRNA levels were measured by Q-RT-PCR on RNA from pupal eye discs dissected from control animals and miR-263a mutants, as well as rescued mutants. There was no significant difference. (0.10 MB TIF) [file pbio.1000396.s008.tif]
