# Supplementary material for: Drosophila microRNAs 263a/b Confer Robustness during Development by Protecting Nascent Sense Organs from Apoptosis
Source: PLoS Biol. 2010 Jun 15;8(6):e1000396. doi: 10.1371/journal.pbio.1000396 (PMC2885982; doi:10.1371/journal.pbio.1000396)
Supplement: Table S1 — List of tested candidate genes, with the corresponding EP lines and results (IOB loss: yes or no) when expression is driven with miR-263b-Gal4 . (0.09 MB DOC) [file pbio.1000396.s009.doc]

**Table S1**: List of tested candidate genes, with the corresponding EP lines and results (IOB loss: yes or no) when expression is driven with *miR-263b-Gal4*.

| Gene name | Name of EP line | IOB loss? |
| --- | --- | --- |
| ab | Bloomington 16949 | no |
| Best2 | Harvard P{XP}d00856 | no |
| br | Bloomington 10858 | no |
| cac | Harvard PBac{WH}f07462 | no |
| CG10208 | Bloomington 16585 | no |
| CG13183 | Bloomington 22192 | no |
| CG14632 | Harvard P{XP}d03688 | no |
| CG30152 | Bloomington 15353 | no |
| CG30217 | Bloomington 22448 | no |
| CG33174 | Bloomington 16986 | no |
| CG3638 | Szeged P{EP}EP964 | no |
| CG3975 | Bloomington 21125 | no |
| CG9238 | Szeged P{EP}EP1040 | no |
| cpo | Szeged P{EP}cpoEP661 | no |
| cycE | Bloomington 4781 | yes |
| DAAM | Harvard PBac{WH}DAAMf06407 | no |
| dacapo | Szeged P{EP}dapEP2584 | no |
| dally | Szeged P{EP}EP3446 | no |
| desert | Harvard PBac{WH}f03377 | no |
| Dg | Szeged P{EP}DgEP2241 | no |
| enc | Bloomington 15539 | no |
| ex | Bloomington 19978 | no |
| fz | Bloomington 19988 | no |
| fz2 | Bloomington 15547 | no |
| G-oα47A | Harvard P{XP}G-oα47Ad02543 | no |
| gap1 | Bloomington 14996 | no |
| Gp150 | Bloomington 20174 | no |
| hid | Harvard P{XP}d10274 | yes |
| how | Bloomington 20038 | no |
| hth | Harvard P{XP}d03358 | no |
| jim | Bloomington 20927 | no |
| Kr | Bloomington 20301 | no |
| kuz | Bloomington 19998 | no |
| LimK | Bloomington 17491 | no |
| Lnk | Harvard PBac{WH}Lnkf05062 | no |
| lola | Bloomington 19872 | no |
| mbl | Szeged P{EP}EP1582 | no |
| mef2 | Bloomington 17230 | no |
| Mes2 | Harvard PBac{WH}Mes2f01255 | no |
| msh6 | Bloomington 16408 | no |
| PGRP-LF | Szeged P{EP}EP3043 | no |
| Ptp99a | Bloomington 16828 | no |
| RhoGAPp190 | Bloomington 20177 | no |
| Sax | Bloomington 15743 | no |
| scrib | Harvard P{XP}d01730 | no |
| Ser | Bloomington 5815 | no |
| skf | Bloomington 20042 | no |
| Slo | Bloomington 18916 | no |
| Smi35a | Harvard P{XP}d01030 | no |
| Snoo | Szeged P{EP}CG7231EP2510 | no |
| sns | Bloomington 17434 | no |
| Spred | Harvard PBac{WH}Spredf03187 | no |
| ssh | Bloomington 16618 | no |
| su(w(a)) | Bloomington 15888 | no |
| tim | Bloomington 15452 | no |
| tws | Harvard P{XP}d05145 | no |
